# Supplementary material for: Single test-based diagnosis of multiple cancer types using Exosome-SERS-AI for early stage cancers
Source: Nat Commun. 2023 Mar 24;14:1644. doi: 10.1038/s41467-023-37403-1 (PMC10039041; doi:10.1038/s41467-023-37403-1)
Supplement: Supplementary file 1 — Suplplementary information [file 41467_2023_37403_MOESM1_ESM.pdf]

Supplementary information

Single test-based early diagnosis of multiple cancer types using Exosome-SERS-AI

Hyunku Shin[1,†], Byeong Hyeon Choi[2,3,†], On Shim[1], Jihee Kim[1], Yong Park[4], Suk Ki Cho[5], Hyun Koo Kim[2,6,*], and Yeonho Choi[1,7,8,9,*]

[1]Exopert corporation, Seoul, 02841, Republic of Korea.

[2]Department of Thoracic and Cardiovascular Surgery, College of Medicine, Korea University Guro Hospital, Seoul 08308, Republic of Korea.

[3]Korea Artificial Organ Center, Korea University, Seoul 02841, Republic of Korea

[4]Division of Hematology-Oncology, Department of Internal Medicine, Korea University College of Medicine, Seoul 02841, Republic of Korea.

[5]Division of Thoracic Surgery, Department of Thoracic and Cardiovascular Surgery, Seoul National University Bundang Hospital, Seongnam 13620, Republic of Korea.

[6]Department of Biomedical Sciences, College of Medicine, Korea University, 02841, Seoul, Republic of Korea

[7]School of Biomedical Engineering, Korea University, Seoul 02841, Republic of Korea.

[8]Department of Biomedical Engineering, Korea University, 02841, Seoul, Republic of Korea

[9]Interdisciplinary Program in Precision Public Health, Korea University, 02841, Seoul, Republic of Korea

†These authors contributed equally.

*Corresponding authors: kimhyunkoo@korea.ac.kr, yeonhochoi@korea.ac.kr

Table of contents

Calculation of SERS Enhancement Factor

Supplementary Fig. 1: Exosome fractionation and marker expression

Supplementary Fig. 2: Preparation of SERS multiarrays

Supplementary Fig. 3: SERS signal enhancement and uniformity

Supplementary Fig. 4: Signal difference

Supplementary Fig. 5: Deep-learning model

Supplementary Fig. 6: Score difference by sex

Supplementary Fig. 7: Comparison with other models in cancer detection

Supplementary Fig. 8: Analysis for architecture and performance

Supplementary Fig. 9: Tissue of origin discrimination model training

Supplementary Fig. 10: Comparison with other models in TOO detection

Supplementary Fig. 11: Decision rules for the integrated model

Supplementary Fig. 12: Prediction for test samples by cancer stage

Supplementary Table 1. Information of clinical subjects

Calculation of SERS Enhancement Factor

To quantify the SERS effect, the analytical enhancement factor (EF) was calculated according to the following equation.

$$EF = \frac{I_{SERS}/C_{SERS}}{I_{RS}/C_{RS}}$$

where C_{SERS} and C_{RS} are the concentration of analytes at the case of SERS and Raman spectroscopy (RS), and I_{SERS} and I_{RS} are the signal intensity at each concentration. In our experiments, all detection setups including laser power, acquisition time, and optical instruments were identical for both conditions. In the RS experiment, signals were detected in a high concentration (C_{RS} = 100 mM) R6G solution to observe a distinguishable peak. I_{RS} at 1364 cm^{-1} was 47.9. In the SERS experiment, signals were detected at a lower concentration (C_{SERS} = 10 μM) to minimize spontaneous Raman activity that could be yielded in an optical path other than the SERS hot-spot. I_{SERS} was 2048.8. Accordingly, the EF was calculated as 4.28 × 10^5.

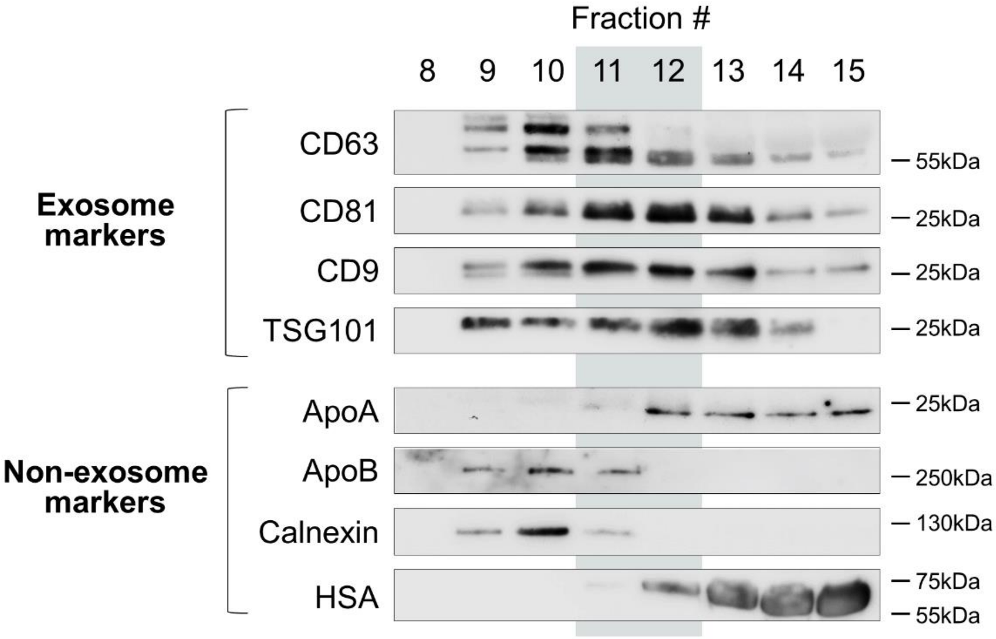

Supplementary Fig. 1: Exosome fractionation and marker expression. CD63, CD81, CD9, and TSG101 are common exosome markers. The non-exosome markers ApoA, ApoB, Calnexin, and human serum albumin (HSA), also were assessed. Fractions 11 and 12 were pooled and used in the diagnostic test. No replicates were performed.

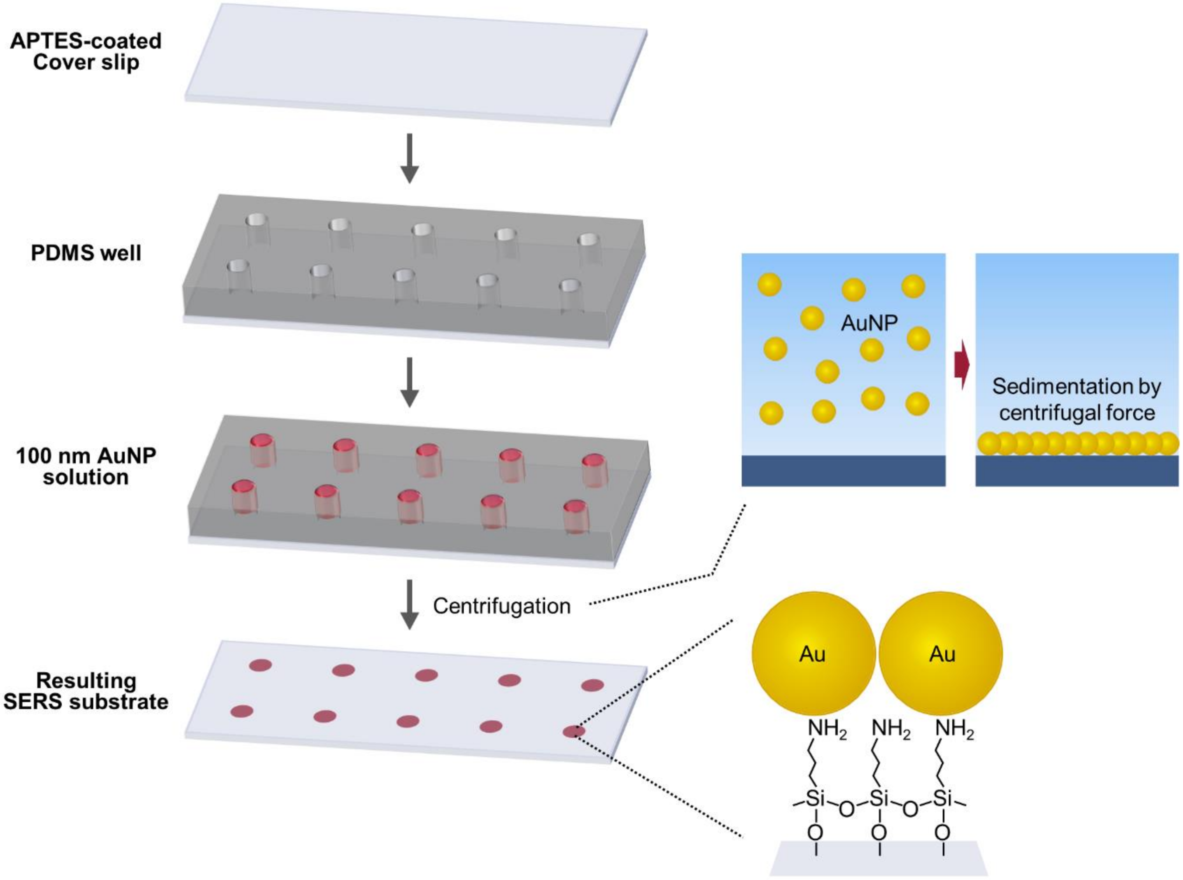

Supplementary Fig. 2: Preparation of SERS multiarrays. A nanoparticle-based hot-spot substrate was fabricated as previously described (Shin et al., Adv. Sci. 7:1903638). In 2.5-mm PDMS well arrays, AuNPs were adhered to the APTES-functionalized cover glass by centrifugation.

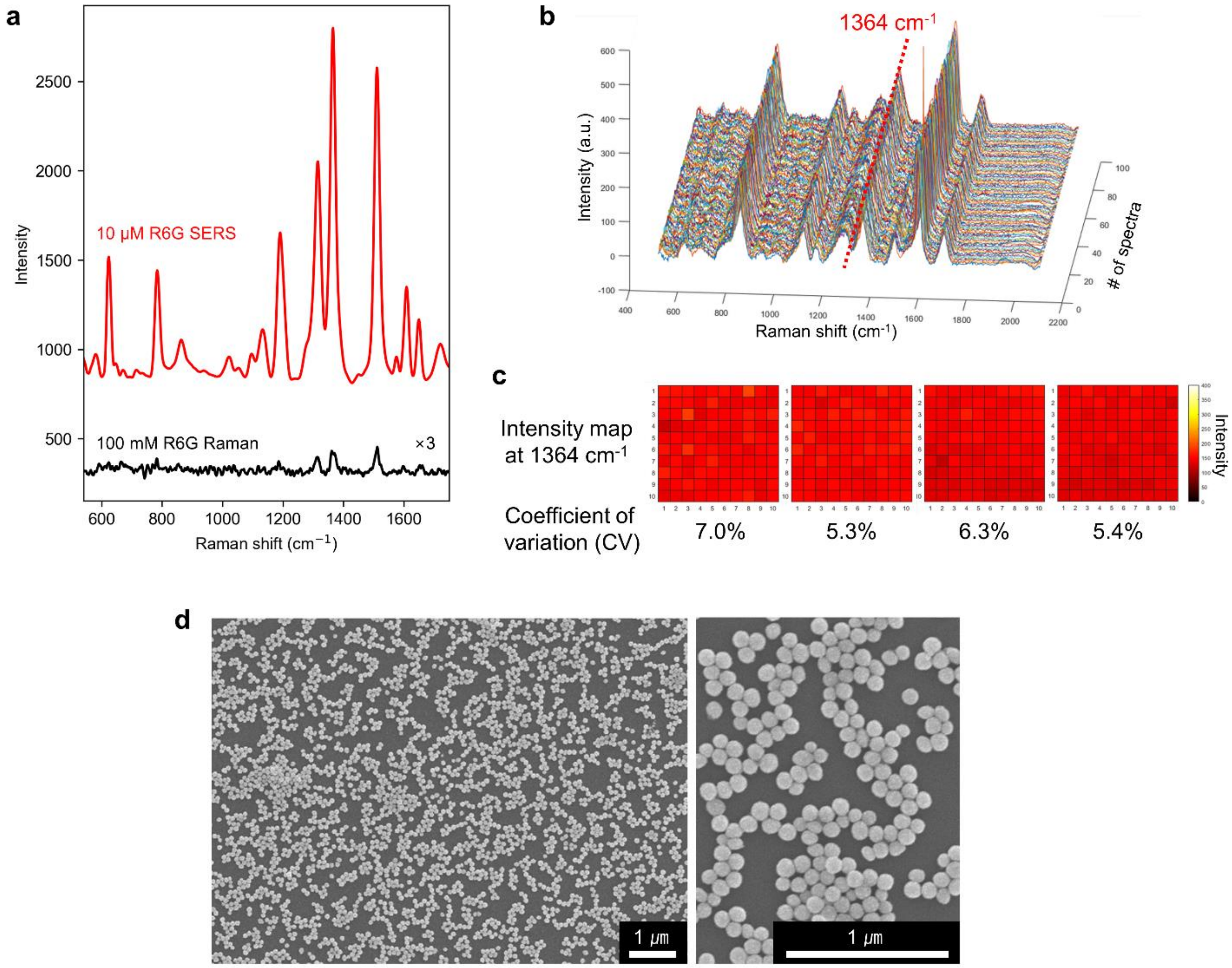

Supplementary Fig. 3: SERS signal enhancement and uniformity. (a) Signal enhancement of the SERS substrate. Each graph represents an averaged spectra of rhodamine 6G (R6G) solution obtained at three different spots. (b) SERS signal uniformity. The SERS signals were scanned after 1 μM of R6G solution was dropped on the array. (c) Intensity maps at 1364 cm^{-1} of the characteristic bands of R6G from 4 repetitive tests. (d) SEM images of the SERS substate. The representative images were cropped from scanned images obtained at different spots over three.

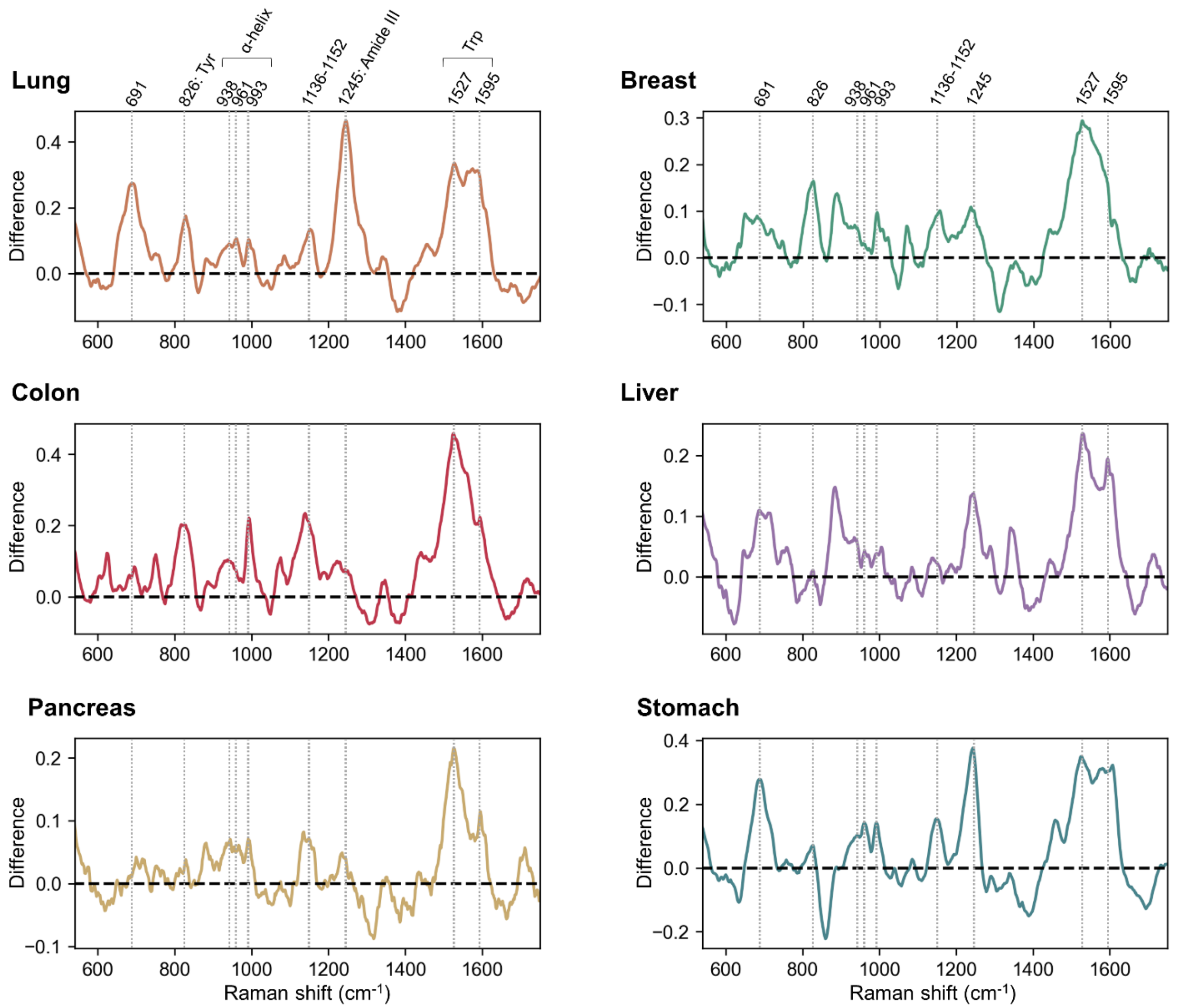

Supplementary Fig. 4: Signal difference. The difference between the average SERS signal of each cancer type and HC is shown. Min-max normalization was performed prior to spectrum subtraction to reduce overall intensity variation. The gray dotted lines indicate common bands detected from all cancer types.

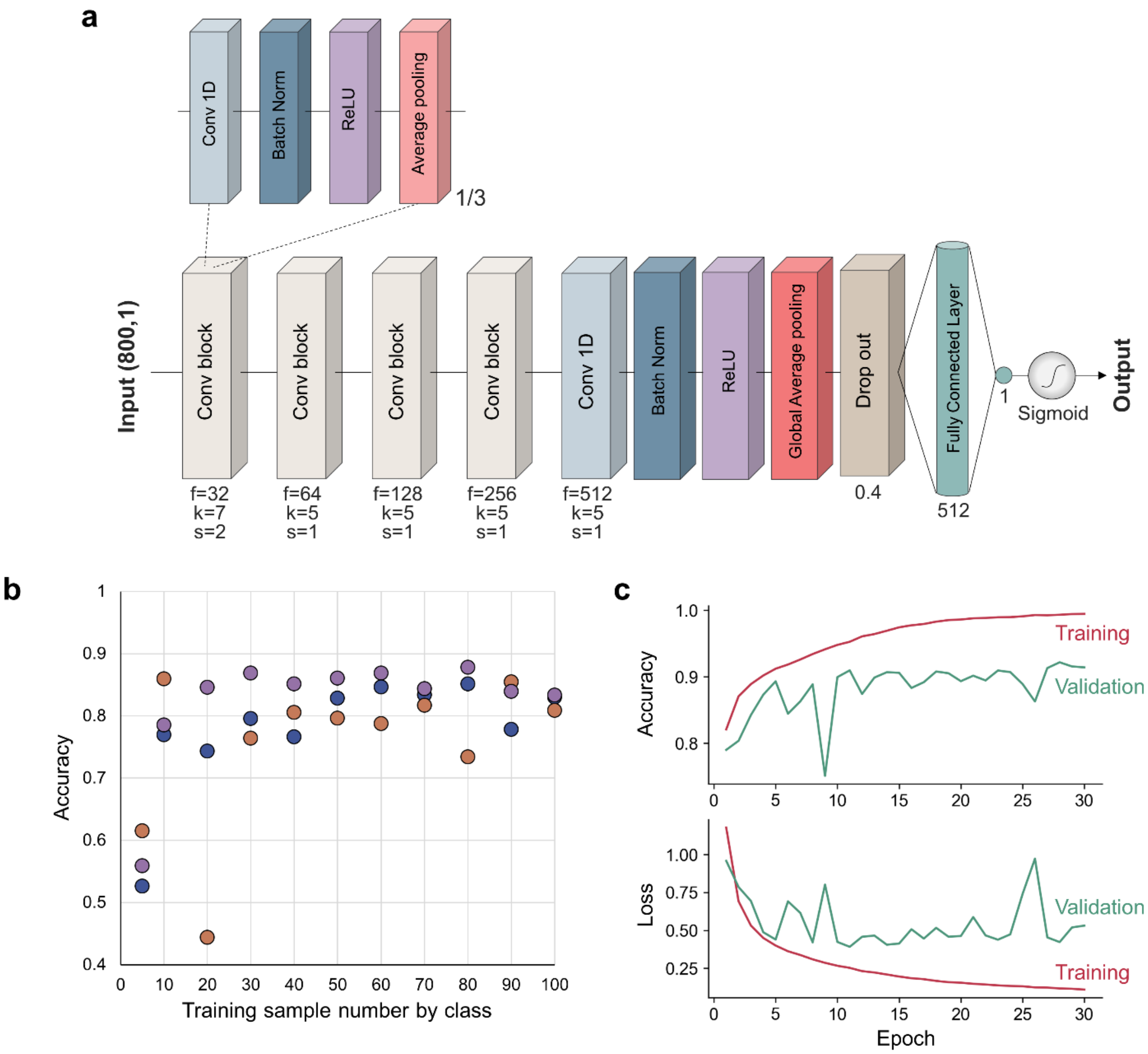

Supplementary Fig. 5: Deep-learning model. (a) Architecture; f, k, and s indicate the numbers of filters, kernel size, and stride size in each convolution layer (Conv 1D). For binary classification, the final activation function was set to be sigmoid. (b) Gradual change in test accuracy with the number of training samples per class. Three repetitions were conducted through random sampling. (c) Loss and accuracy curves from the implementation of the cancer diagnosis model. The RMSprop optimizer was utilized in the learning step with a learning rate of 0.00035, a decay rate of 0.000181, and a batch size of 32.

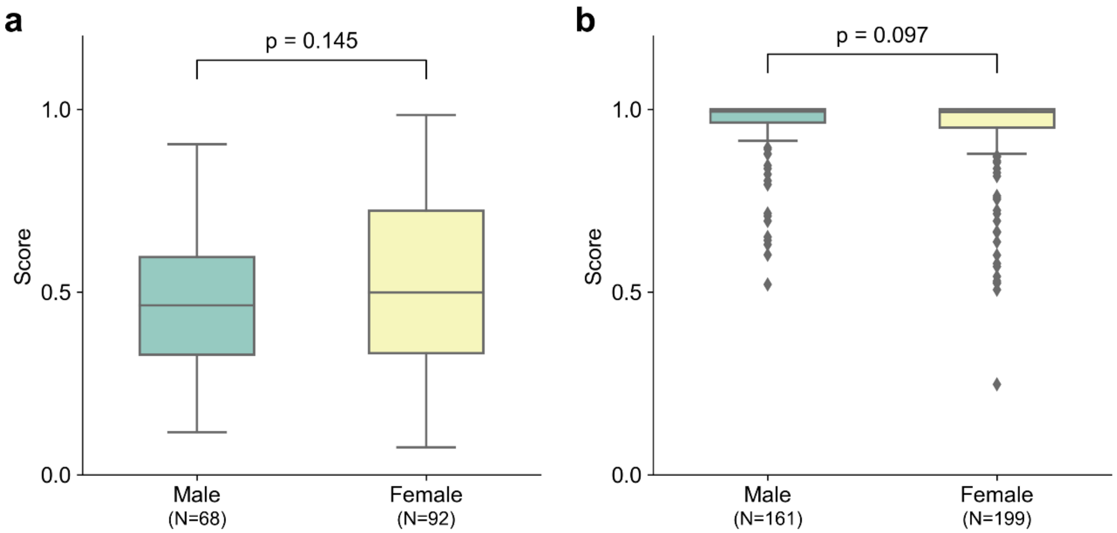

Supplementary Fig. 6: Score difference by sex. Two-sided t-tests were performed to examine statistical differences. (a) Healthy controls. The 95% confidence intervals, effect sizes, and degrees of freedom are [-0.12~0.02], 0.228, and 155.7, respectively. (b) Cancer patients. The 95% confidence intervals, effect sizes, and degrees of freedom are [-0~0.04], 0.176, and 358, respectively. The central line, box, errorbar, and dots indicate the median, inter-quartile range (Q1 and Q3), min-max range, and outliers, respectively.

	Dummy	SVM	CNN
CV1	0.722	0.789	0.810
CV2	0.722	0.797	0.823
CV3	0.721	0.784	0.790
CV4	0.723	0.781	0.788
CV5	0.721	0.787	0.810

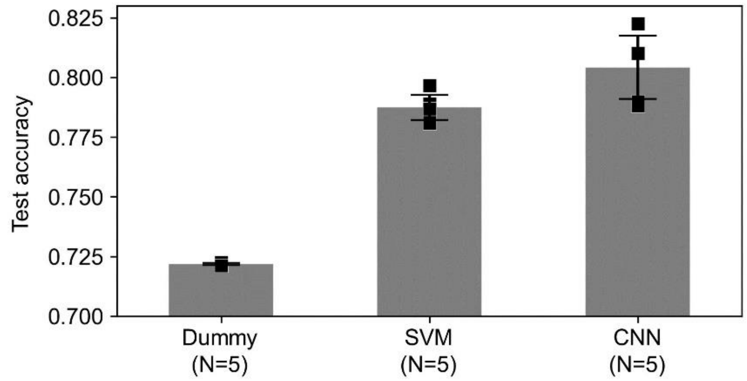

Supplementary Fig. 7: Comparison with other models in TOO detection. As a baseline model, dummy classifier and support vector machine (SVM) were established. The accuracy represents data-wise prediction accuracy for the test dataset in each model. Errorbars indicate the standard deviation.

a

Layer	Layer type	Parameter
	Input	
1	Conv1D<br>Batch normalization<br>Activation (ReLU)<br>Average pooling	Filter size 1
2	Conv1D<br>Batch normalization<br>Activation (ReLU)<br>Average pooling	Filter size 2
3	Conv1D<br>Batch normalization<br>Activation (ReLU)<br>Average pooling	Filter size 2
4	Conv1D<br>Batch normalization<br>Activation (ReLU)<br>Average pooling	Filter size 2
5	Conv1D<br>Batch normalization<br>Activation (ReLU)	Filter size 2
6	Global average pooling	
7	Dropout	Dropout rate
	Flatten	
8	Dense	FCL size 1
9	Dense	FCL size 2
	Output	

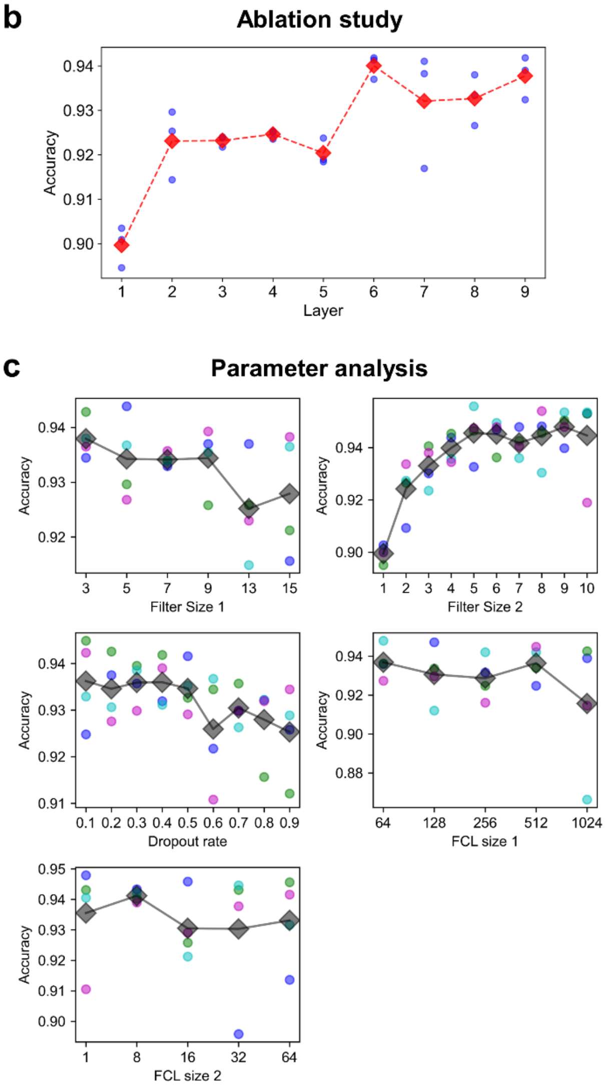

Supplementary Fig. 8: Analysis for architecture and performance. (a) Layer and parameter designation. (b) Ablation study to investigate the accuracy change as each layer is removed from the intact network architecture. (c) Parameter analysis. Validation accuracy according to filter size, dropout rate, and fully-connected layer (FCL) size in the network learning process. In each analysis, parameters except a variable were fixed at 7 (filter size 1), 5 (filter size 2), 0.4 (dropout rate), 512 (FCL size 1), and 1 (FCL size 2).

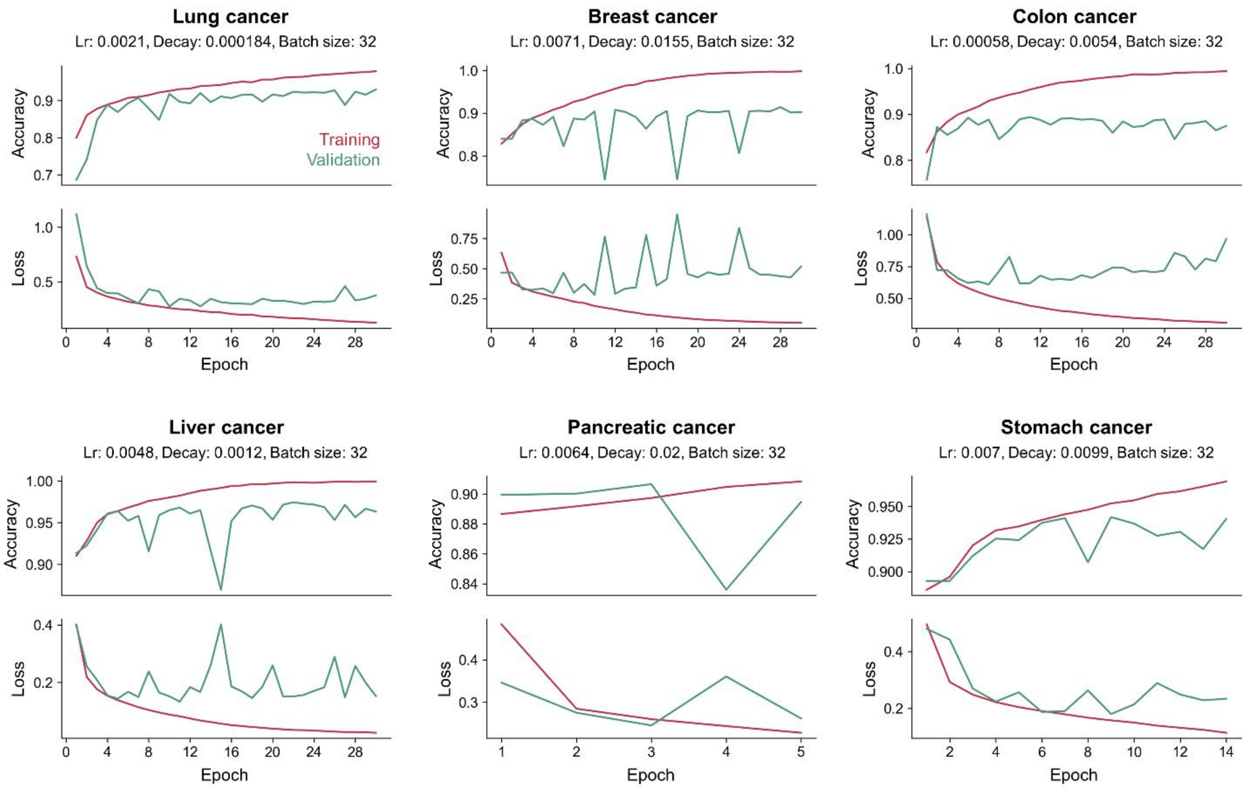

Supplementary Fig. 9: Tissue of origin discrimination model training. Accuracy and loss curves for each cancer type. The RMSprop optimizer was utilized in the learning step with the displayed learning rate (Lr), decay rate, and batch size.

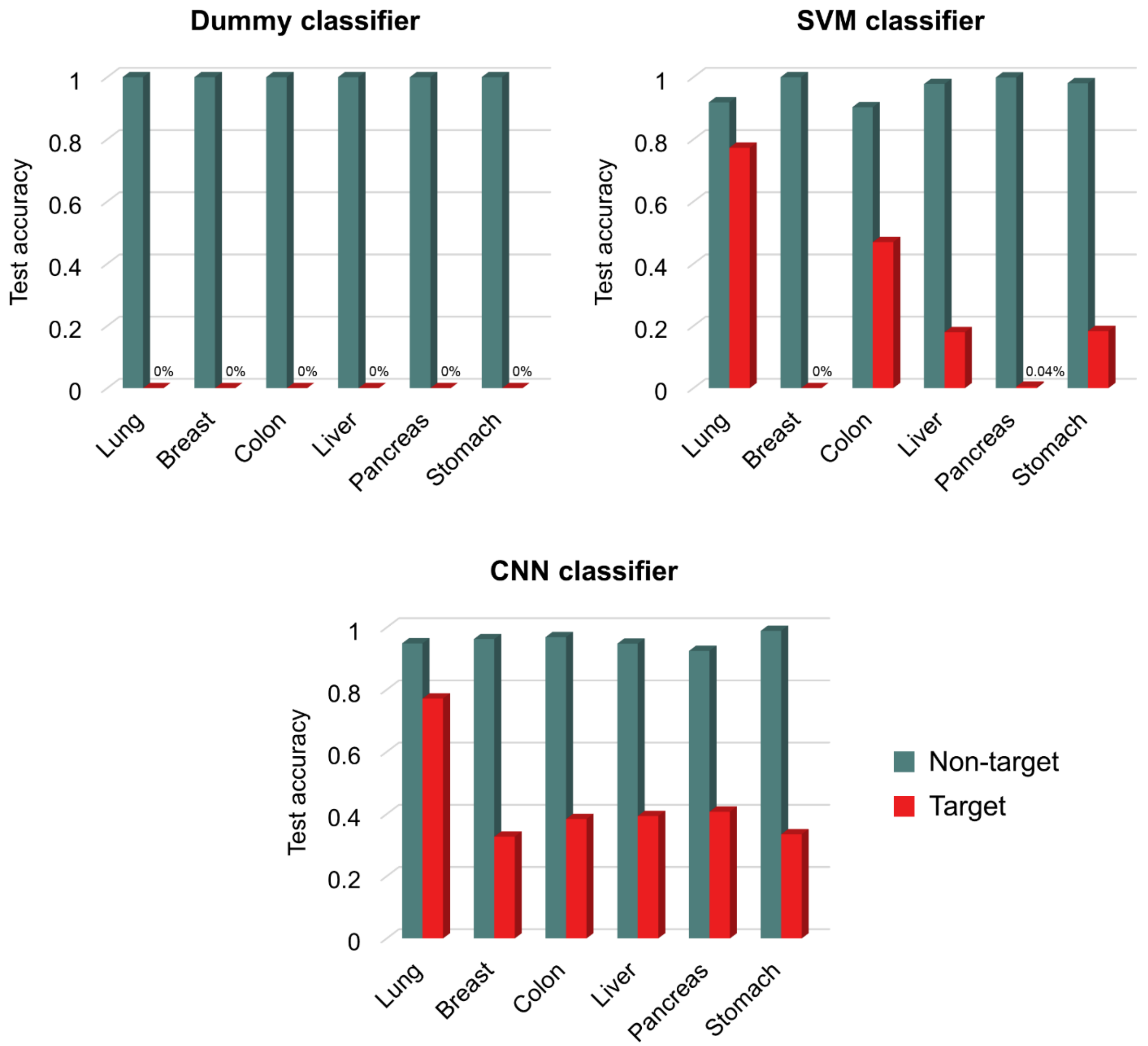

Supplementary Fig. 10: Comparison with other models in TOO detection. As a baseline model, dummy classifier and support vector machine (SVM) were established. The accuracy represents data-wise prediction accuracy for the test dataset in each model. In cases where data were overly biased, percentages were displayed.

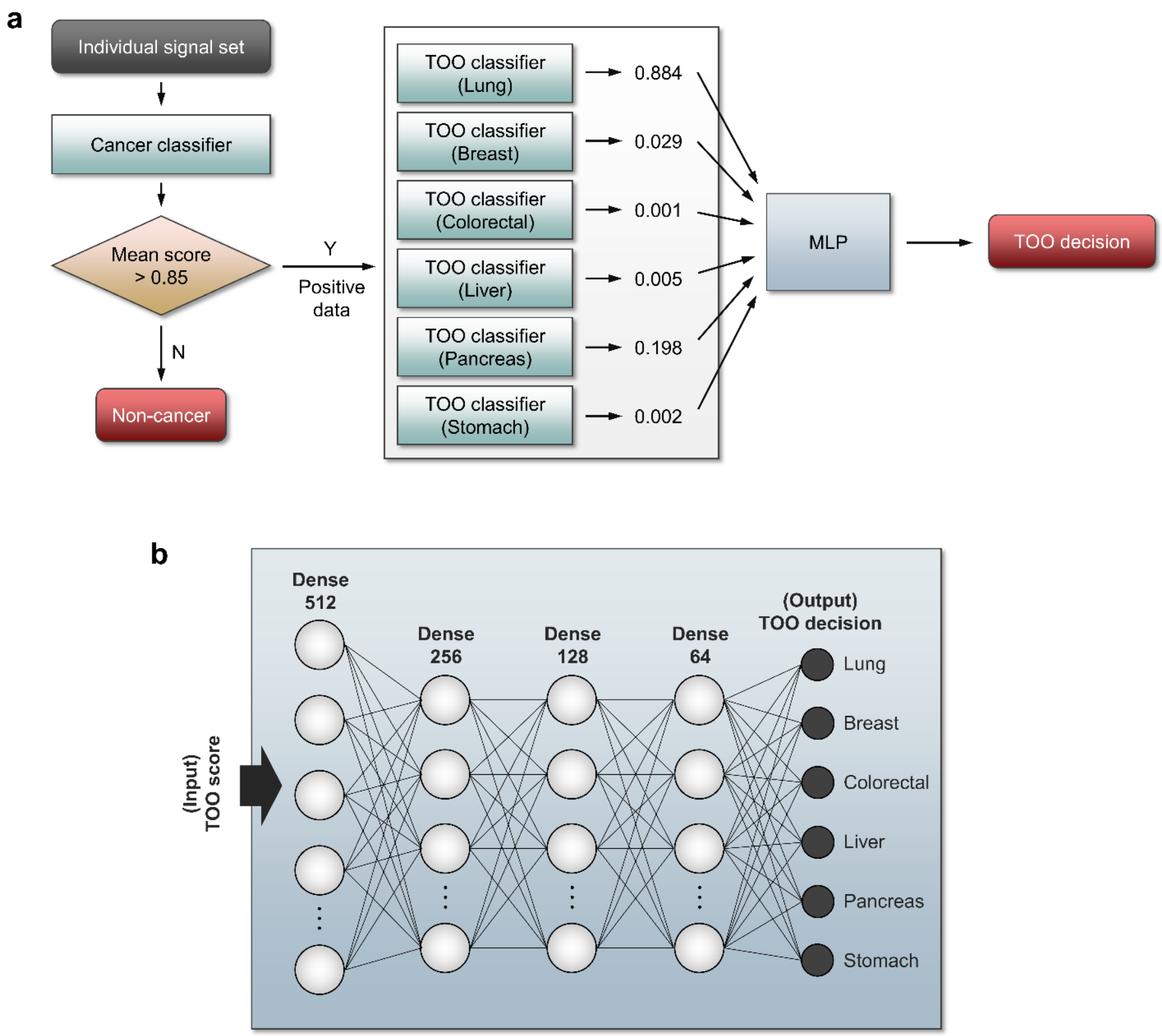

Supplementary Fig. 11: Decision rules for the integrated model. (a) Decision flow chart. (b) Architecture of multi-layer perceptron (MLP) model to determine TOO of a sample. The MLP model was implemented using TOO score predicted from training samples.

a

HC vs Early-stage cancer

Actual class		Predicted class: Non-cancer	Lung cancer	Breast cancer	Colon cancer	Liver cancer	Pancreatic cancer	Stomach cancer
	Non-cancer	**151**	1	0	3	0	4	1
	Lung cancer	24	**63**	0	0	1	2	1
	Breast cancer	3	0	**40**	5	3	12	6
	Colon cancer	4	0	1	**31**	3	4	3
	Liver cancer	2	2	1	0	**8**	3	0
	Pancreatic cancer	0	1	0	1	2	**26**	2
	Stomach cancer	0	4	2	0	0	0	**18**

Actual class	Predicted class: Pos	Neg	
**Pos**	245	33	**Sensitivity** 0.881
**Neg**	9	151	**Specificity** 0.944

b

HC vs Advanced cancer

Actual class		Predicted class: Non-cancer	Lung cancer	Breast cancer	Colon cancer	Liver cancer	Pancreatic cancer	Stomach cancer
	Non-cancer	**151**	1	0	3	0	4	1
	Lung cancer	0	**8**	1	0	0	0	0
	Breast cancer	0	0	**0**	0	0	1	0
	Colon cancer	2	0	0	**13**	0	6	2
	Liver cancer	0	2	3	1	**11**	6	1
	Pancreatic cancer	0	0	0	2	0	**3**	1
	Stomach cancer	0	2	1	0	1	1	**11**

Actual class	Predicted class: Pos	Neg	
**Pos**	77	2	**Sensitivity** 0.975
**Neg**	9	151	**Specificity** 0.944

Supplementary Fig. 12: Confusion matrix for test samples by cancer stage. (a) Early-stage cancer detection. (b) Advanced cancer detection.

Supplementary Table 1. Information of clinical subjects

Characteristics			Number	Percentage (%)
**Healthy control**	Sex	Male	93 (44.3)	
		female	117 (55.7)	
	Age	Mean ± SD	54 ± 6	-
		Range	40 ~ 75	
**Lung cancer**	Sex	Male	69 (43.9)	42/58
		female	88 (56.1)	
	Age	Mean ± SD	64 ± 8	-
		Range	40 ~ 86	
	Cancer type	Adenocarcinoma	157	100
	Pathological TNM stage	TisN0M0	5	3.2%
		T1a(mi)N0M0	15	9.6%
		T1aN0M0	19	12.1%
		T1bN0M0	22	14.0%
		T1cN0M0	20	12.7%
		T1cN2M0	2	1.3%
		T2aN0M0	43	27.4%
		T2aN1M0	8	5.1%
		T2aN2M0	5	3.2%
		T2bN0M0	11	7.0%
		T2bN2M0	1	0.6%
		T2N1M0	1	0.6%
		T2N2M0	1	0.6%
		T3N0M0	1	0.6%
		T3N1M0	1	0.6%
		T3N2M0	1	0.6%
		T4N1M0	1	0.6%
**Breast cancer**	Sex	female	100 (100%)	
	Age	Mean ± SD	50 ± 10	-
		Range	34 ~ 82	
	Cancer type	Infiltrating duct carcinoma	100	100
	Pathological TNM stage	T1miN0M0	1	1.0%
		T1aN0M0	4	4.0%
		T1bN0M0	14	14.0%

		T1bN1miM0	1	1.0%
		T1cN0M0	22	22.0%
		T1cN1aM0	4	4.0%
		T1cN1miM0	1	1.0%
		T1N0M0	30	30.0%
		T1N1M0	4	4.0%
		T2N0M0	9	9.0%
		T2N1aM0	4	4.0%
		T2N1M0	3	3.0%
		T2N2aM0	1	1.0%
		T2N2M0	2	2.0%
**Colon cancer**	Sex	Male	64 (58.2)	65/35
		female	46 (41.8)	
	Age	Mean ± SD	63 ± 10	-
		Range	34 ~ 85	
	Cancer type	Adenocarcinoma	110	100
	Pathological TNM stage	T1N0M0	7	6.4%
		T1N1aM0	1	0.9%
		T2N0M0	9	8.2%
		T2N1aM0	2	1.8%
		T2N1bM0	2	1.8%
		T2N1M0	1	0.9%
		T2N2aM0	1	0.9%
		T3N0M0	52	47.3%
		T3N0M1b	1	0.9%
		T3N1aM0	7	6.4%
		T3N1bM0	4	3.6%
		T3N1bM1a	1	0.9%
		T3N1cM0	1	0.9%
		T3N2aM0	6	5.5%
		T3N2aM1a	3	2.7%
		T3N2bM0	4	3.6%
		T4aN1aM0	1	0.9%
		T4aN1bM1c	1	0.9%
		T4aN2aM1c	2	1.8%
		T4aN2bM1b	1	0.9%
		T4bN0M0	1	0.9%

		n/a	2	1.8%
**Liver cancer**	Sex	Male	46 (82.1)	89.7/10.3
		female	10 (17.9)	
	Age	Mean ± SD	57 ± 10	-
		Range	36 ~ 78	
	Cancer type	Hepatocellular carcinoma	56	100
	BCLC STAGE	Very early stage (0)	10	17.9%
		Early stage (A)	10	17.9%
		Intermediate stage (B)	19	33.9%
		Advanced stage (C) or Terminal stage (D)	17	30.4%
	Tumor size (cm)	Mean ± SD, range	4.00 ± 3.76, 1.00 ~17.80	-
**Pancreatic cancer**	Sex	Male	29 (48.3)	48.3/51.7
		female	31 (51.7)	
	Age	Mean ± SD	65 ± 9	-
		Range	45 ~ 86	
	Cancer type	Infiltrating duct carcinoma	60	100
	Pathological TNM stage	T1N0M0	3	5.0%
		T1N1M0	1	1.7%
		T1N2M1	1	1.7%
		T1cN0M0	5	8.3%
		T1cN1M0	1	1.7%
		T2N0M0	13	21.7%
		T2N1M0	20	33.3%
		T2N2M0	4	6.7%
		T3N0M0	6	10.0%
		T3N1M0	2	3.3%
		T3N2M0	2	3.3%
		n/a	2	3.4%
**Stomach cancer**	Sex	Male	41 (68.3)	80/20
		female	19 (31.7)	
	Age	Mean ± SD	61 ± 11	-
		Range	39 ~ 81	

Cancer type	Adenocarcinoma	60	100
Pathological TNM stage	T1aN0M0	5	8.3%
	T1bN0M0	11	18.3%
	T1N2M0	1	1.7%
	T2N0M0	9	15.0%
	T2N1M0	1	1.7%
	T2N3M0	1	1.7%
	T3N0M0	4	6.7%
	T3N0M1	1	1.7%
	T3N1M0	1	1.7%
	T3N2M0	1	1.7%
	T3N3aM0	1	1.7%
	T3N3bM0	1	1.7%
	T3N3M0	2	3.3%
	T4aN0M0	4	6.7%
	T4aN2M0	3	5.0%
	T4aN3aM0	1	1.7%
	T4bN0M0	1	1.7%
	T4bN2M0	1	1.7%
	T4bN3bM0	2	3.3%
	T4N2M0	1	1.7%
	T4N2M1	1	1.7%
	T4N3M0	5	8.3%
	T4N3M1	2	3.3%

206
