# Supplementary material for: Single test-based diagnosis of multiple cancer types using Exosome-SERS-AI for early stage cancers
Source: Nat Commun. 2023 Mar 24;14:1644. doi: 10.1038/s41467-023-37403-1 (PMC10039041; doi:10.1038/s41467-023-37403-1)
Supplement: Supplementary file 3 — Reporting Summary [file 41467_2023_37403_MOESM3_ESM.pdf]

nature portfolio

Corresponding author(s): Hyun Koo Kim, Yeonho Choi

Last updated by author(s): Feb 20, 2023

Reporting Summary

Nature Portfolio wishes to improve the reproducibility of the work that we publish. This form provides structure for consistency and transparency in reporting. For further information on Nature Portfolio policies, see our Editorial Policies and the Editorial Policy Checklist.

Statistics

For all statistical analyses, confirm that the following items are present in the figure legend, table legend, main text, or Methods section.

n/a	Confirmed	
☐	☒	The exact sample size (*n*) for each experimental group/condition, given as a discrete number and unit of measurement
☐	☒	A statement on whether measurements were taken from distinct samples or whether the same sample was measured repeatedly
☐	☒	The statistical test(s) used AND whether they are one- or two-sided<br>*Only common tests should be described solely by name; describe more complex techniques in the Methods section.*
☒	☐	A description of all covariates tested
☒	☐	A description of any assumptions or corrections, such as tests of normality and adjustment for multiple comparisons
☐	☒	A full description of the statistical parameters including central tendency (e.g. means) or other basic estimates (e.g. regression coefficient) AND variation (e.g. standard deviation) or associated estimates of uncertainty (e.g. confidence intervals)
☐	☒	For null hypothesis testing, the test statistic (e.g. *F*, *t*, *r*) with confidence intervals, effect sizes, degrees of freedom and *P* value noted<br>*Give P values as exact values whenever suitable.*
☒	☐	For Bayesian analysis, information on the choice of priors and Markov chain Monte Carlo settings
☒	☐	For hierarchical and complex designs, identification of the appropriate level for tests and full reporting of outcomes
☒	☐	Estimates of effect sizes (e.g. Cohen's *d*, Pearson's *r*), indicating how they were calculated

Our web collection on statistics for biologists contains articles on many of the points above.

Software and code

Policy information about availability of computer code

Data collection	For acquisition of Raman spectral data, Andor solis (v4.31) was utilized to operate the spectrometer system.
Data analysis	The classification algorithms in this paper were implemented and operated using custom Python code with TensorFlow (v2.5), numpy (v1.23), and pandas (v1.5) packages. To calculate the diagnostic results (e.g. ROC curves, sensitivity, specificity) and statistical results, custom codes implemented using Python libraries such as scikit-learn (v1.0) and pingouin (v0.5) were utilized. In order to draw the graphs, seaborn (v0.12) and matplotlib (v3.5) python libraries, and Matlab (R2021a) were utilized. The codes to predict test samples will be shown in GitHub public repository (https://github.com/Hyunkushin/OTMC_NatComm).

For manuscripts utilizing custom algorithms or software that are central to the research but not yet described in published literature, software must be made available to editors and reviewers. We strongly encourage code deposition in a community repository (e.g. GitHub). See the Nature Portfolio guidelines for submitting code & software for further information.

Data

Policy information about availability of data

All manuscripts must include a data availability statement. This statement should provide the following information, where applicable:

- Accession codes, unique identifiers, or web links for publicly available datasets
- A description of any restrictions on data availability
- For clinical datasets or third party data, please ensure that the statement adheres to our policy

Source data constituting the graph in this study are provided as an Excel file with this paper.

Human research participants

Policy information about studies involving human research participants and Sex and Gender in Research.

Reporting on sex and gender	In this study, clinical samples were collected based on biological sex. Sex of participants was defined based on self-report. A total of 342 male and 411 female clinical samples were used in this study. The detailed information on sex by cancer types is available in the supplementary information.
Population characteristics	Inclusion criteria of this research include 1) An adult of Korean nationality, 2) Patients who received cancer surgery and permanent pathology of lung cancer (adenocarcinoma), breast cancer (duct carcinoma), colon cancer (adenocarcinoma), liver cancer (hepatocellular carcinoma), pancreatic cancer (duct carcinoma), and stomach cancer (adenocarcinoma). 3) Patients without neoadjuvant therapy before cancer surgery, and 4) Patients who have not been diagnosed with other cancers before cancer surgery. The plasma samples were collected before surgery after a permanent pathology was confirmed. Exclusion criteria is patients who do not meet the inclusion criteria.
Recruitment	The eligible biospecimen of cancer patients were retrospectively, randomly obtained, and prospectively collected clinical data according to inclusion/exclusion criteria through 3 human biobanks (Biobank of Korea University Guro Hospital, Asan Bio-Resource Center, and Biobank of Ajou University Hospital) in the Republic of Korea. Blood plasma samples from HCs without a personal cancer history were retrospectively, randomly obtained from the Korea Institute of Radiological and Medical Sciences (KIRAMS) Radiation Biobank, and the Biobank of Seoul National University Bundang Hospital, Republic of Korea. Sex of participants was defined based on self-report. We obtained informed consent from all participants who underwent blood collection.
Ethics oversight	The Institutional Review Board of Korea University Guro Hospital approved this study (approvals 2020GR0176 and 2021GR0013 for the recruitment and collection of HC subjects and cancer patients, respectively).

Note that full information on the approval of the study protocol must also be provided in the manuscript.

Field-specific reporting

Please select the one below that is the best fit for your research. If you are not sure, read the appropriate sections before making your selection.

☒ Life sciences ☐ Behavioural & social sciences ☐ Ecological, evolutionary & environmental sciences

For a reference copy of the document with all sections, see nature.com/documents/nr-reporting-summary-flat.pdf

Life sciences study design

All studies must disclose on these points even when the disclosure is negative.

Sample size	Since this study was a pilot study to develop a method to identify multiple cancers is possible, no sample size calculation to explore clinical utility was performed. Considering the number of samples required for training models, we tried to verify the proposed method through at least dozens of test samples for each cancer type. All available samples that met the inclusion criteria from each biobank were used in the study; thus, a total of 753 blood plasma samples were used.
Data exclusions	No data were excluded.
Replication	The western blot result and TEM images in fig. 2 were collected from at least two independent experiments. In western blots in supplementary fig. 1, replication was not performed because this result is simply to show an example of the operation result of the previously examined exosome isolation technology (Sci Rep 11, 217 (2021). https://doi.org/10.1038/s41598-020-80514-8).<br>Prediction through the AI system was derived using 100 data per subject.
Randomization	The eligible biospecimen of cancer patients and healthy controls were retrospectively, randomly collected from biobanks.<br>Train samples to implement the algorithm and test samples to validate the algorithm were randomly selected using Python random library.<br>The data for training were shuffled in advance.

Blinding	The study to implement AI models was conducted with open-label because actual labels were required to train models. However, the training samples and test samples were clearly separated. The predictions on the test sample were made without information on the correct class.

Reporting for specific materials, systems and methods

We require information from authors about some types of materials, experimental systems and methods used in many studies. Here, indicate whether each material, system or method listed is relevant to your study. If you are not sure if a list item applies to your research, read the appropriate section before selecting a response.

Materials & experimental systems

n/a	Involved in the study	
☐	☒	Antibodies
☒	☐	Eukaryotic cell lines
☒	☐	Palaeontology and archaeology
☒	☐	Animals and other organisms
☐	☒	Clinical data
☒	☐	Dual use research of concern

Methods

n/a	Involved in the study	
☒	☐	ChIP-seq
☒	☐	Flow cytometry
☒	☐	MRI-based neuroimaging

Antibodies

Antibodies used	CD9 (santacruz, sc13118, mouse monoclonal, D2420); CD63 (Bioss, bs-1523R, Rabbit polyclonal, AH09126615); CD81 (santacruz, sc166028, mouse monoclonal, A0220); TSG101 (biorbyt, orb576823, Rabbit polyclonal, AS5455); ApoA1 (abcam, ab227455, Rabbit polyclonal, N/A); ApoB (abcam, ab20737, Rabbit polyclonal, N/A); Calnexin (abcam, ab22595, Rabbit polyclonal, N/A); Humanserum albumin (Bioss, bs-0945R, Rabbit polyclonal, 990294W). Some lot numbers could not be identified because the antibodies were discarded after use. The dilution rate for western blotting is described in the methods section.
Validation	https://www.scbt.com/ko/p/cd9-antibody-c-4<br>https://www.biossusa.com/products/bs-1523r<br>https://www.scbt.com/p/cd81-antibody-d-4?requestFrom=search<br>https://www.biorbyt.com/tsg101-antibody-orb576823.html<br>https://www.abcam.com/apolipoprotein-a-i-antibody-ab227455.html<br>https://www.abcam.com/apolipoprotein-b-antibody-ab20737.html<br>https://www.abcam.com/calnexin-antibody-er-marker-ab22595.html<br>https://www.biossusa.com/products/bs-0945r

Clinical data

Policy information about clinical studies

All manuscripts should comply with the ICMJE guidelines for publication of clinical research and a completed CONSORT checklist must be included with all submissions.

Clinical trial registration	The Institutional Review Board of Korea University Guro Hospital approved this study (approvals 2020GR0176 and 2021GR0013 for HC subjects and cancer patients, respectively). (ClinicalTrials.gov identifier: NCT04529915)
Study protocol	The details about this study is described at methods section.
Data collection	Since this study used retrospectively collected samples, the period of time for recruitment and data collection was not established. Clinical data including biological sex, birth date, disease history, smoking history, alcohol history, pathological stage, and tumor lesion information were collected.
Outcomes	1. 2020GR0176<br>1-A. Primary Outcome Measures :<br>Evaluation of the distinction between healthy controls and lung cancer patients through deep-learning analysis of exosomes [Time Frame: 3 years]<br>Comparative evaluation of whether it is possible to distinguish between healthy controls and lung cancer patients through deep-learning analysis of exosomes<br>Evaluating the possibility of distinguishing between normal and lung cancer patients through the analysis of lung cancer-specific exosomal protein [Time Frame: 3 years]<br>Quantitative analysis using lung cancer-specific exosomal protein evaluated the possibility of distinguishing between healthy controls and lung cancer patients.<br>1-B. Secondary Outcome Measures :<br>Evaluation of the possibility of distinguishing the early pathological stages in lung cancer patients through deep-learning analysis of exosomes [Time Frame: 3 years]<br>Evaluating whether the early stages of lung cancer patients can be distinguished using deep-learning analysis of exosomes<br>Evaluation of the possibility of distinguishing the early pathological stages in lung cancer patients through quantitative analysis of lung cancer specific exosomal proteins [Time Frame: 3 years]<br>Evaluating whether the early stages of lung cancer patients can be distinguished using quantitative analysis of lung cancer specific exosomal proteins

2. 2021GR0013
2-A. Primary Outcome Measures :
Evaluation of the distinction between healthy controls and various cancer patients through deep-learning analysis of exosomes
Evaluating whether the pathological stages of various cancer patients can be distinguished using deep-learning analysis of exosomes
Evaluation of the distinction between healthy controls and various cancer patients through exosome protein analysis of exosomes
Evaluation of the possibility of distinguishing the pathological stages in various cancer patients through quantitative and statistical analysis
2-B. Secondary Outcome Measures :
Evaluation of the possibility of distinguishing the early pathological stages in various cancer patients through deep-learning analysis of exosomes
Evaluation of the possibility of distinguishing the early pathological stages in various cancer patients through quantitative analysis of exosomal proteins
Evaluating whether the early stages of cancer patients can be distinguished using quantitative and statistical analysis
